# Are Methionine Sulfoxide-Containing Proteins Related to Seed Longevity? A Case Study of *Arabidopsis* *thaliana* Dry Mature Seeds Using Cyanogen Bromide Attack and Two-Dimensional-Diagonal Electrophoresis

**DOI:** 10.3390/plants11040569

**Published:** 2022-02-21

**Authors:** Ewa Marzena Kalemba, Benoît Valot, Dominique Job, Christophe Bailly, Patrice Meimoun

**Affiliations:** 1 Institute of Dendrology, Polish Academy of Sciences, Parkowa 5, 62-035 Kórnik, Poland; kalemba@man.poznan.pl; 2 UMR 7622 Biologie du Développement, IBPS, Sorbonne Université, CNRS, F-75005 Paris, France; christophe.bailly@sorbonne-universite.fr; 3 PAPPSO, INRA, CNRS, AgroParisTech, Université Paris-Saclay, GQE-Le Moulon, 91190 Gif-sur-Yvette, France; benoit.valot@univ-fcomte.fr; 4 UMR CNRS 6249 Chrono-Environnement, Université de Bourgogne Franche-Comté, 25000 Besançon, France; 5 UMR5240, CNRS, Université Claude Bernarnard Lyon 1, INSA, Bayer CropScience, 69622 Lyon, France; job.dominique@gmail.com

**Keywords:** methionine sulfoxide, mass spectrometry, oxidative stress, post-translational modifications, protein modification, redox proteomics, two-dimensional diagonal electrophoresis, seed viability

## Abstract

In recent years, several reports pointed out the role of protein oxidation in seed longevity, notably regarding the oxidation of methionine (Met) residues to methionine sulfoxide (MetO) in proteins. To further consider this question, we present a handy proteomic method based on the use of two-dimensional diagonal electrophoresis (2Dd) and cyanogen bromide (CNBr) cleavage, which we refer to as 2Dd-CNBr. CNBr treatment of proteins causes the non-enzymatic hydrolysis of peptide bonds on the carboxyl side of reduced Met residues. However, Met oxidation causes a lack of cleavage, thus modifying the electrophoretic mobility of CNBr-induced peptides. This approach was first validated using bovine serum albumin as a model protein, which confirmed the possibility of distinguishing between oxidized and non-oxidized forms of Met-containing peptides in gels. Then, the 2Dd-CNBr method was applied to the *Arabidopsis thaliana* seed protein extract in a control (non-oxidized) condition and in an oxidized one (as obtained following hypochlorous acid treatment). Twenty-four oxidized Met residues in 19 proteins identified by mass spectrometry were found to be surface exposed in these proteins. In the three-dimensional environment of the oxidized Met, we detected amino acid residues that could be converted by oxidation (carbonylation) or by phosphorylation, suggesting a possible interplay between Met oxidation and the other protein modifications. The identification of the proteins oxidatively modified in Met residues revealed the finding that MetO-containing proteins are related to seed longevity. Based on these results, we suggest that the method presently described also has the potential for wider applications.

## 1. Introduction

Despite their potentially harmful reactivity toward most biomolecules, reactive oxygen species (ROS) also play key signaling roles in many biological processes in a wide range of organisms [1]. Endogenously generated ROS, produced by electron transport chains, react with different macromolecules in the cell and cause oxidative damage. Such oxidative damage accumulates over time during the life cycle of many organisms, and has been suggested to be one possible cause of aging [2]. In the plant kingdom, ROS and redox signaling are major cellular actors in various events throughout the plant life, from growth and development to stress responses [3,4,5,6]. Protein oxidation is assumed to be an important process affecting seed germination and vigor [7,8]. Furthermore, mRNAs and proteins stored in the dry mature seeds, as well as selective translation from stored mRNAs, are key features of seed germination [9,10]. Therefore, plant seeds constitute an excellent system for studying protein oxidation and for developing novel technical approaches in this field of proteome research. The role of ROS in seed science has emerged in the last decade and it is now well established that a regulated accumulation of ROS, which is referred to as the oxidative window for germination [11,12], is a prerequisite for radicle elongation, the first visible symptom of seed germination [11].

Due to their abundance and their chemical composition, proteins constitute a major target for ROS, especially concerning the amino acids cysteine (Cys) and methionine (Met), whose side chains contain a sulfur atom [13,14,15]. The oxidation of Met first leads to Met sulfoxide (MetO) formation, a redox type of post-translational modification. This reaction can be reversed through the specific action of methionine sulfoxide reductases (MSR) A (EC number 1.8.4.11) and B (EC number 1.8.4.12), acting on *S*- and *R*-diastereomers of MetO, respectively [16]. Then, a more severe oxidative attack of Met results in the irreversible formation of a sulfone derivative (Met sulfone) [17]. It is surmised that the oxidation of proteins is not necessarily a deleterious phenomenon, and in vivo, the reversibility of methionine sulfoxidation suggests a key role of this protein modification in the regulation of cellular mechanisms [18,19,20,21].

The MSR system is indeed found in all organisms, from bacteria to humans, and the consequences of Met redox changes are well documented in mammals [22,23,24]. The enzymatic regeneration of MetO through MSR activity encompasses three types of mechanisms [25]: (i) the regulation of signaling pathways, (ii) the action of Met as an antioxidant for protecting proteins from higher oxidative events causing damage in protein function [26], and (iii) the subsequent formation of protein-carbonyl adducts [27]. However, little information is available on the nature of MSR substrates, notably concerning the biochemical and structural features involved in Met oxidation in proteins, mainly due to the difficulty in isolating these oxidized protein targets. The plant MSR system is well described and its role has been documented in response to environmental constraints, during development, and aging, and particularly in the tolerance to oxidative stress [28,29]. Importantly, the MSR system has been shown to control seed longevity in *Medicago truncatula* and *Arabidopsis thaliana* (herein referred to as Arabidopsis) [30]. MSR isoforms were investigated in many plant species, and their roles were revealed from studies examining the effects of external factors as well as mutants (for a review, see Rey and Tarrago [28]). Several redox proteomic approaches, including affinity chromatography, enabled the identification of MSR partners among MetO-containing proteins. In this way, 24 protein partners of the Arabidopsis plastidial MSRB1 were identified in the leaf extracts [31]. Unstressed leaves from various plant species were shown to contain 2–6% MetO, while this value further increased to more than 50% under stress-inducing environmental conditions [32,33,34]. In contrast, MetO levels in dry mature seeds are about 30% and decrease upon seed imbibition and the completion of germination [35].

Global analyses of methionine oxidation were performed predominantly in human proteome, thus revealing that proteins containing MetO exist even at unstressed conditions [36]. They disclosed that specific amino acid sequences, protein types, and cellular localization favor Met oxidation. More precisely, disordered proteins are highly prone to oxidation in cell extracts [37], and nuclear as well as mitochondrial proteins are preferentially oxidized inside living cells [38]. Additionally, the oxidation of Met residues within phosphorylation motifs proved to be a highly selective phenomenon among stress-related proteins [39]. Among global MetO plant studies, only Arabidopsis catalase 2 knockout plants were characterized in detail, identifying nearly 400 proteins with mainly an oxidoreductase activity, involved in the response to abiotic and biotic stimulus, and located primarily in the chloroplasts [40].

Consistent with a role in aging [30,41], several recent studies have pointed out the role of protein oxidation in seed longevity, notably including oxidation at Met residues in proteins [42,43,44]. To further document this question and toward a global characterization of oxidation of Met residues in proteins to MetO, we have developed a handy proteomic method called 2Dd-CNBr based on the use of two-dimensional diagonal electrophoresis (2Dd) and cyanogen bromide (CNBr) cleavage after the first dimension [45], followed by mass spectrometry for the identification of proteins of interest. The CNBr reagent hydrolyzes Met residue C-terminal polypeptide bond with a high specificity. However, the presence of an alcohol residue (Thr and Ser) after Met or the modification of Met, e.g., by oxidation, hinders cleavage [46,47,48]. CNBr cleavage has already been successfully used in gel and it allows for the analysis by mass spectrometry of the C-terminal sequences of proteins in *Shewanella oneidensis* [49]. On this basis, this novel method relies on the lack of cleavage after CNBr treatment when protein Met is oxidized, contrarily to reduced Met residues that are attacked by CNBr yielding homoserine (HS) or homoserine lactone. In this work, this method allowed us to successfully identify 24 in vivo oxidized Met sites within 19 different Arabidopsis seed proteins. An examination of these proteins confirmed the finding that MetO-containing proteins are related to seed longevity. Finally, our successful validation of the 2Dd-CNBr method in Arabidopsis seeds opens the possibility of analyzing other protein extracts at a global level and/or for a targeted use.

## 2. Results

### 2.1. A Novel Proteomic Approach: 2Dd-CNBr

We have developed a novel method coupling 2Dd using CNBr cleavage after the first protein electrophoresis and mass spectrometry to allow evidencing the oxidation of Met residues in proteins, at a high throughput, and on complex mixtures of proteins. The method is based on the fact that CNBr can hydrolyze peptide bonds at the C-terminus of only non-oxidized Met residues, whereas oxidized Met does not react with CNBr [46,47]. In this technique, CNBr cleavage is carried out between two SDS-PAGE separations, allowing the separation of CNBr cleavage products in the second dimension. 

**Figure 1:**
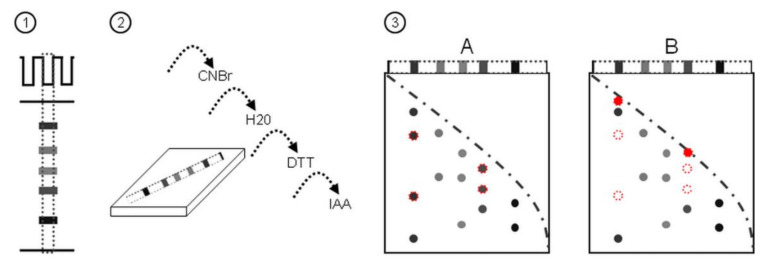
Schematic representation of the 2Dd strategy using CNBr cleavage after the first electrophoretic dimension and presentation of the expected results. (**1**) SDS-PAGE of protein mixtures; (**2**) the gel strip is cut out and incubated with agitation for 30 min in CNBr cleavage buffer at room temperature (approx. 22 °C). The gel strip is rinsed briefly in deionized water and washed twice for 5 min in water. Then, the gel strip is equilibrated twice for 10 min in an equilibration solution containing dithiothreitol (DTT) and iodoacetamide (IAA); (**3**), the gel strip is placed on top of acrylamide gel to perform a second-dimensional separation by SDS-PAGE. (**A**) Control condition (reduced); (**B**) oxidizing condition; red dotted circles and red circles represent reduced and oxidized form of proteins, respectively.

**Figure 2:**
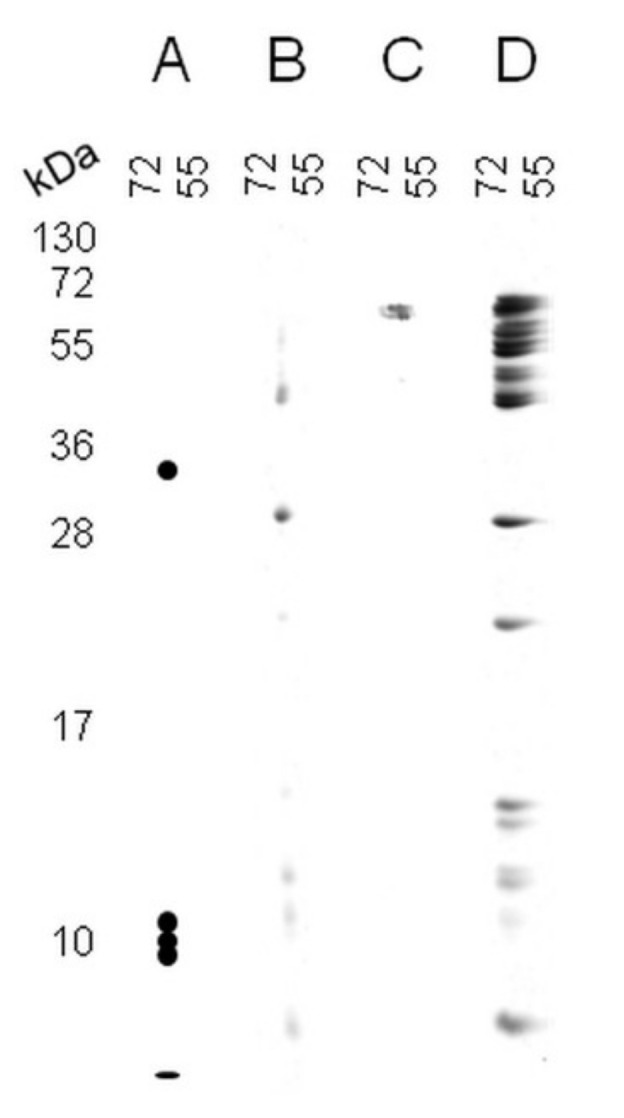
2Dd-CNBr analysis of BSA. (**A**) Theoretical profile of fully non-oxidized BSA after 2Dd-CNBr; (**B**) experimental profile obtained with 100 ng BSA in non-oxidizing conditions (control); (**C**) experimental profile obtained with 100 ng BSA oxidized by HOCl in gel; (**D**) experimental profile obtained with 10 µg BSA in the non-oxidizing condition. Proteins were visualized by silver staining. Molecular mass markers are indicated for both dimensions.

Figure 1 shows a synoptic scheme of the 2Dd-CNBr strategy together with the theoretical results expected that permit differentiating the Met oxidation status of proteins from comparisons of 2Dd-CNBr profiles. To evaluate the experimental potential of this approach, the technique was first conducted using BSA as a model protein to highlight the possibility to discriminate the reduced and oxidized states of Met residues in gel. BSA (607 amino acids) was used because it contains only few Met residues (at positions 1, 87, 184, 445, and 547) with a homogenous repartition in the amino acid sequence [50]. Theoretically, if all Met residues contained in BSA were reduced, CNBr attack should yield five digest products (Figure 2A). Published protocols generally use a large molar excess of CNBr over Met residues for efficient CNBr cleavage [46,51]. This finding was assessed by using different CNBr concentrations, ranging from 50 mM to 1 M, during different treatment durations, from 10 min to 90 min, and with different BSA quantities from 50 ng (0.75 pmole) to 10 µg (0.15 nmole). The obtained results are consistent with the view that an excess of CNBr over Met favors CNBr cleavage (Figure 2).

Figure 2B shows the results obtained when 100 ng BSA (1.5 pmoles) was subjected to 2Dd-CNBr with the use of 0.5 M CNBr for 30 min. In these conditions, the BSA protein proved to be entirely digested by CNBr, as no protein band could be revealed by silver staining in the MW range of 55–72 kDa, corresponding to the native molecular weight of BSA (66 kDa). In contrast, when a 100-fold higher amount of BSA protein (10 μg) was used under the same 2Dd-CNBr conditions (0.5 M CNBr, 30 min), CNBr attack was not efficient, as a large part of the protein was found near 66 kDa by SDS-PAGE (Figure 2D). In gel, the oxidation of BSA in the presence of 0.5 mM HOCl (Figure 2C) prior to CNBr treatment revealed that oxidized BSA was not cleaved in these conditions. As HOCl directly oxidizes Met to MetO [52], all Met residues in BSA were converted to MetO, resulting in the absence of cleavage products in the 2Dd-CNBr profile, as expected. Based on these results, we conclude that the 2Dd-CNBr method allows for deciphering the protein Met oxidation status in the BSA model protein.

### 2.2. 2Dd -CNBr of Arabidopsis Seed Soluble Proteins

**Figure 3:**
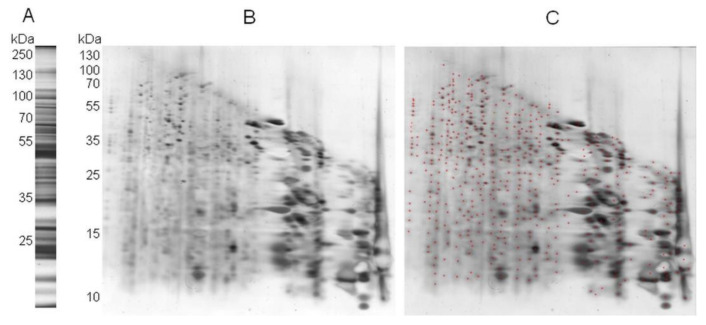
1D and 2Dd-CNBr analyses of 40-µg soluble protein extracts from mature Arabidopsis seeds. (**A**) 1D separation of Arabidopsis seed proteins; (**B**) 2Dd-CNBr (control non-oxidized condition). Proteins sre visualized by silver staining; (**C**) as in (**B**), where red crosses indicate detected spots by using Image Master Platinum software (GE Healthcare, Chicago, IL, USA). Molecular mass markers are indicated for both dimensions.

We then used the 2Dd-CNBr approach using complex mixtures of proteins, as extracted from mature Arabidopsis seeds. As in all proteomic analyses, the preparation of protein extracts is a critical step. Met is highly susceptible to oxidation, and experimental extraction procedures can lead to artificial oxidation of proteins. To avoid such artifactual oxidation of Met, we performed a fast protein extraction in the presence of DTT as the reducing agent, as described in the experimental procedures section. After grinding the mature Arabidopsis seeds in liquid nitrogen, proteins were precipitated with 10% (*v*/*v*) TCA and then solubilized in a Laemmli buffer [53], quantified, and loaded in SDS-PAGE to perform the 2Dd-CNBr. The 1D-SDS-PAGE analysis of the proteins obtained after such a fast protein extraction is presented in Figure 3A. Figure 3B shows a representative 2Dd-CNBr profile of soluble proteins extracted from the Arabidopsis seeds. More than 500 spots corresponding to CNBr attack products were revealed by silver staining at a wide range of molecular weights, ranging from 130 kDa to less than 10 kDa (Figure 3C).

**Figure 4:**
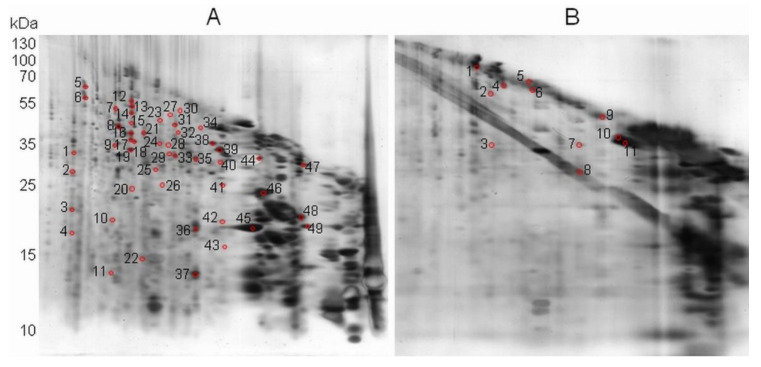
2Dd-CNBr analysis of 40-µg soluble protein extracts from mature Arabidopsis seeds, in control (non-oxidized) and oxidized (HOCl) conditions. (**A**) 2Dd-CNBr (control non-oxidized condition); (**B**) 2Dd-CNBr (in gel oxidized proteins). Proteins are visualized by silver staining. Molecular mass markers are indicated for both dimensions. Red marks represent proteins analyzed by mass spectrometry.

In marked contrast, when the proteins were oxidized in gel with 0.5 mM HOCl prior to the CNBr treatment, as was done for BSA (Figure 2C), much less digestion products were revealed and many of the proteins migrated at the gel diagonal (Figure 4B).

As with the BSA protein (Figure 2), we conclude that our approach allows for the discrimination of the protein Met oxidation status at the proteome scale by the comparison of control and oxidized profiles. To further validate the 2Dd-CNBr approach, 60 spots from 2Dd-CNBr gels were analyzed by mass spectrometry. Spots were collected from proteins with different abundances (i.e., corresponding to major and minor bands in 1D-SDS PAGE) and in a wide range of molecular weights (e.g., from 15 to 210 kDa). Mass spectrometry analysis of spots from control non-oxidized conditions (Figure 4A, spot no 1 to 49) led to the identification of 986 peptides, with 77 peptides containing at least one Met residue (Table S1). 

Among them, 20 peptides contained at least one Met residue exhibiting reduced, oxidized (increased mass of +15.99491 Da corresponding to MetO), or modified forms (−29.9928 Da and −48.0034 Da corresponding to homoserine and homoserine lactone, respectively). The 20 peptides led to the identification of 11 proteins (Table S2) that can be considered as abundant proteins, as they were identified from major spots (Figure 4A, spot no. 36–39 and 44–49) resulting from major protein bands obtained after 1D-SDS PAGE. As observed with BSA (Figure 2D), the efficiency of the CNBr attack was related to the abundance of the target proteins. Thus, the presence of reduced Met in these spots suggests that CNBr attack was not fully effective, because in the case of a complete CNBr cleavage, all reduced Met residues should have been modified in homoserine or homoserine lactone. These results further confirmed the need to use CNBr in large excess relative to the protein amounts. Hence, the results concerning major spots could not safely be taken into account and were not considered in the present analysis. The data showed that among 704 experimental peptides, 29 peptides contained an oxidized Met and 23 a modified Met (Table S3). For five oxidized peptides, a Thr or a Ser residue was found after a Met residue. In such cases, the CNBr cleavage was not efficient, as shown previously [46,47,48], and therefore, in this case, no firm conclusion could be made regarding the accuracy of the oxidation state of Met. Then, 24 oxidized Met were evidenced in 25 experimental peptides, leading to the identification of 19 proteins (Table 1 and Table S4). Moreover, the analysis of spots from HOCl-inducing oxidizing conditions (Figure 4B, spot no 1 to 11) led to identifying 378 peptides, of which 110 contained at least one Met with an increased mass of +15.99491 Da (Table S5). In this case, the identification of exclusively oxidized Met was consistent with the changes observed in the 2Dd-CNBr profile in oxidized conditions (Figure 4B).

**Figure 5:**
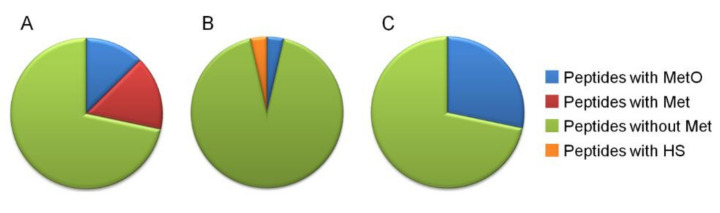
Methionine content analysis of identified peptides by mass spectrometry after 1D SDS-PAGE (**A**), 2Dd-CNBr carried out in control (non-oxidized) (**B**) and oxidized (HOCl) (**C**) conditions. (**A**) 5968 peptides were identified by two independent mass spectrometry analyses; 1348 peptides contained at least one methionine including 757 and 1109 oxidized and reduced Met residues, respectively (see Table S6). (**B**) 704 peptides were identified and 29 contained at least an oxidized (+15.99491 Da) and 23 a modified Met residue (−29.9928 Da or −48.0034 Da) (see Table S3). (**C**) 378 peptides were identified and 110 contained at least one oxidized Met residue (+15.99491 Da) (see Table S5). HS, homoserine.

A comparison of the Met content in peptides identified by mass spectrometry after 1D-SDS-PAGE (Figure 5A), 2Dd-CNBr in control (Figure 5B), and oxidized (HOCl) (Figure 5C) conditions allowed for the validation of the present results. After 1D electrophoresis, spots collected from proteins distributed in a range of molecular weights from 15 kDa to 210 kDa were submitted to mass spectrometry analyses. From these analyses, 5968 peptides were identified (Table S6), among which 22.58% contained at least one Met (Figure 5A). In 2Dd-CNBr carried out in the control conditions (Figure 5B), all reduced Met from non-abundant spots were attacked by CNBr and modified into homoserine or homoserine lactone, as expected. Hence, it can be concluded that 24 identified oxidized Met reported in the 19 Arabidopsis seed proteins (Table 1 and Table S4) were oxidized in vivo.

**Figure 6:**
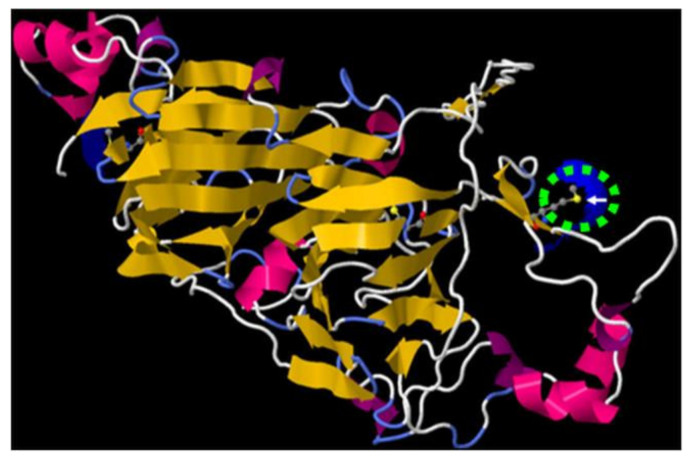
3D model of Arabidopsis CRA1 protein (AT5G44120). The model was built using Jmol software. The white arrow in the green dotted circles show the oxidized Met position; blue areas show solvent-accessible surface.

WebLogo 3.3 was used to create sequence logos to graphically represent common features of the primary sequences surrounding these 24 oxidized Met residues (Figure S1). These analyses did not allow for evidencing specific amino acid sequences promoting Met oxidation. However, further analyses on a larger number of proteins are needed to more precisely explore this question. Importantly, three-dimensional (3D) models available for 14 identified Arabidopsis proteins (Table S7) showed that the 17 identified oxidized Met present in these proteins were surface exposed (Figure 6 and Figures S2–S14), further supporting the finding that surface-exposed Met residues in proteins are particularly sensitive to oxidation [21,31,54,55]. The 3D environment of identified oxidized Met residues (within a distance of 7 Å, which corresponds to the radius where amino acids may influence others [45]) was analyzed to evidence a 3D environment favorable for Met oxidation. The most striking feature is that the nearest amino acids (<3 Å) to the oxidized Met were mostly hydrophobic. When increasing the distance from the oxidized Met, the proportion of hydrophobic amino acids decreased, whereas the proportion of hydrophilic amino acids increased (Figure S15). The oxidation of Met could lead to the modification of the surface hydrophobicity in proteins, and could result in a local change of folding [56,57]. In this context, Met oxidation could interact with other types of amino acid modifications by increasing or decreasing the accessibility of certain residues. 

In the neighborhood of all of the identified MetOs, we searched for the presence of amino acids known to be potentially modified, such as Pro, Arg, and Glu, or Ser, Thr, and Tyr in the case of carbonylation or phosphorylation, respectively. Among the identified Arabidopsis proteins, five proteins (AT2G42560.1, AT1G21750.1, AT5G44120.3, AT5G52300.1, and AT2G36530.1) were previously identified as being carbonylated in Arabidopsis seeds [58]. Two Arabidopsis proteins, AT1G21750.1 and AT5G44120.3, contained amino acids considered sensitive to carbonylation in the vicinity of the identified oxidized Met in 3D models (AT1G21750.1: Pro142, Glu184; AT5G44120.3: Glu114, Arg136). Met sulfoxidation can also interact with the phosphorylation of the target proteins. We observed that seven of the presently identified proteins that contained MetO (AT1G07920: M264, AT1G21750: M180, AT4G12290: M491, AT5G44120: M138, AT5G20960: M1046, AT1G54100: M251, and AT1G47710: M15) also contained a phosphorylation site in the close vicinity of MetO in the 3D structure (Table 2). All these results document a broad applicability of the 2Dd-CNBr method to finely analyze the interplay between Met oxidation and other post-translational modifications of proteins. 

## 3. Discussion

Throughout plant life, plants are subjected to several biotic and abiotic stresses. In response, plants induce complex signaling networks, including ROS signaling. Protein oxidation thus underpins many cellular processes, and the characterization of the oxidized protein targets is essential in order to better understand the regulations operating during redox signaling. Met sulfoxide and MSRs are known to play roles in these pathways. For example, the role of the MSR system was demonstrated to affect seed longevity in *Medicago truncatula,* Arabidopsis [30] and *Fagus sylvatica* [41]. Recently, it was revealed that the abundance of MSRB2 was related to seed desiccation tolerance [60]. Yet, despite the biological importance of this process, there is a lack of a reliable and simple method for the characterization of oxidized Met in proteins due to the difficulty to isolate and identify this protein modification. 

Except COmbined FRActional DIagonal Chromatography (COFRADIC) proteomics technology used for comparing wild-type Arabidopsis and *catalase 2* mutant [40] plants, recent papers describe the use of shotgun proteomics with ^18^O-labeled hydrogen peroxide and redox-Met specific probes in humans [36] and bacteria [61], or with methionine bioconjugation [62,63]. A gel-free CNBr-based method was described to investigate MetO in peptides containing two Met residues [64]. Our approach presented here, 2Dd-CNBr (Figure 1), is a novel method enabling studies of in vivo Met oxidation in proteins and peptides with no limits to the number of Met residues. In our opinion, this method will be very effective for the analysis of complex mixtures of proteins to visualize and further analyze proteins of interest. We believe that the use of 2Dd-CNBr can deliver new opportunities and novel results in different research areas compared to methods only based on CNBr cleavage [46,47,48,49], based on CNBr cleavage and electrophoresis of one purified protein [65], or based on diagonal electrophoresis without CNBr cleavage [14,45]. This is because in our approach, CNBr-cleavage is performed in gel on a complex mixtures of proteins. Diagonal electrophoresis is useful for investigations of the subunit composition of complex proteins [35], as well as intermolecular and intramolecular disulfide bridges in redox variations of this procedure [66]. The use of 2Dd may be useful for the comparison of samples representing multiple conditions to identify proteins, for which the electrophoretic migration pattern is altered via their redox state to eventually focus the analysis on these proteins. The 2Dd-CNBr approach is a fast and easy-to-use method with a high reproducibility. Furthermore, one of the economic advantages of 2Dd-CNBr is a low sample cost in comparison with the current protein mass spectrometry and proteomics service fees. The interpretation of 2Dd maps includes all spots stained with silver nitrate. Although this type of staining provides limited sensitivity for a qualitative approach, the 2Dd-CNBr method appears not to be affected by the presence of major proteins in seed protein extracts (e.g., CRU), which might have masked the identification of proteins less represented in MS. Thus, the results clearly reveal protein candidates for more detailed analyses. Although the selection and identification of certain proteins from 2Dd maps will not reach the efficacy of high-throughput analyses, the 2Dd-CNBr method enables avoiding puzzling biological responses in the numerous data collected after an omics approach.

In the present work, this method was first standardized and validated using BSA as a model protein (Figure 2). The results highlighted the power of this tool to discriminate between reduced and oxidized Met through the comparison of the electrophoretic profiles. Applying 2Dd-CNBr to Arabidopsis seed proteins allowed for the identification, through mass spectrometry, of 24 oxidized Met present in 19 proteins (Table 1 and Table S4). An analysis of 3D models available for 14 out of the 19 identified Arabidopsis proteins disclosed that Met exposure at the protein surface predominantly favors its oxidation, in agreement with previous studies [21,31,54,55,67]. The data also suggest that the local amino acid sequence surrounding Met residues has no substantial effect on oxidation, in agreement with previous work based on the identification of oxidized Met in peptides and proteins by COFRADIC [40,54]. However, additional experiments considering larger numbers of proteins containing oxidized Met residues are necessary to further confirm this point. Considering that Met oxidation can lead to a modification of the surface hydrophobicity of affected proteins and in a local change of folding [56,57], it can be proposed that reversible Met oxidation is an important modulator of protein function, activity, stability, or structure. In this respect, we note that the presently identified protein targets are known to play important roles in seed biology (Table 1 and Table S4). 

Several late embryogenesis abundant (LEA) proteins confer desiccation tolerance to plant seeds [68,69,70,71]. Increased sensitivity of LEA proteins to oxidative conditions might be a result of their lack of tertiary structure, as they exhibit a large degree of intrinsic disordered 3D structure [72], a feature in agreement with the finding that surface exposition favors Met oxidation in proteins (Figure 6 and Figures S2–S14) [21,31,54,55,67] and with the fact that intrinsically disordered proteins are highly prone to oxidation [37]. Similarly, the identified oxidized Met (M138) was found located in a disordered part of Arabidopsis CRA1 (Table 1, Figure 6 and Figure S16). These disordered regions participate in the control of the quaternary structures of cruciferins in a hexameric form found in the protein storage vacuoles of seeds (PSVs) [73]. Therefore, our results suggest that the oxidation state of the Met (M138) residue might be involved in the stabilization of the quaternary structure of this storage protein. For example, in the case of seed storage protein oxidation by carbonylation, it has been proposed that as this PTM increases the susceptibility of proteins to proteolytic cleavage [73,74,75,76], the observed carbonylation of 12S-cruciferin subunits occurring during seed development might facilitate their mobilization during germination [58]. In addition, it has been shown that the redox state of Met controls phase transitions predominantly in proteins with low-complexity domains [74]. More precisely, the sulfoxidation of Met residues in yeast ataxin-2 transformed this protein from liquid-like droplets to labile polymers, whereas the MSRs action restored the liquid-like state [74]. Cruciferin displays conformational flexibility [75]. The conformational changes of the proteins induced by aspartic peptidases are required for the dense packaging of seed storage proteins within PSVs [76]. In this context, MetO might induce structural changes in 12S seed storage proteins, and could interfere with their packaging during seed maturation and their mobilization during seed germination. Importantly, these changes might be reversible, because MSRs were recently immunolocalized in PSVs [41]. Among the MetO-containing proteins identified in our study (Table 1), protein disulfide isomerase conferring redox homeostasis and Elongation factor 1 playing a role in protein expression, were identified as being altered in *MsrA* and *MsrB1* mutants of human embryonic kidney cells, respectively, exposed to oxidative stress [23]. Elongation factor 1 and aspartyl protease (Table 1) are both involved in translation. Several studies highlighted the importance of the translation machinery in conditioning seed longevity and vigor, more precisely, in aging of Arabidopsis [7] and pea seeds [77], and in germination of Arabidopsis [9] and rice seeds [78]. In Arabidopsis, loss of seed vigor during aging correlates with the carbonylation of disulfide isomerases [7], which are essential folding catalysts and chaperones of the ER [79]. Serpins (Table 1) are involved in the regulation of programmed cell death [80], which was initiated from the onset of aging in pea seeds and eventually led to seed viability loss [77]. Aldehyde dehydrogenases that remove aldehydes formed by oxidative stress [81], and 4-Coumarate:CoA ligase (EC 6.2.1.12) involved in seeds in the biosynthesis of flavonoids and therefore in regulation of dormancy or viability [82,83], are other proteins essential for the maintenance of seed viability [84]. Both above mentioned proteins were identified as targets of Met oxidation in the present work (Table 1). Last, but not least, mitochondrial dihydrolipoyl dehydrogenase (DLD; EC 1.8.1.4) administrating alpha-lipoic acid is beneficial to a number of diseases caused by oxidative stress in animals [85,86], and regulates the lifespan and aging in yeasts [87] and pea seeds [77]. MetO-containing proteins identified in our study (Table 1) involved aldehyde oxidase and indole-3-acetaldehyde oxidase, two enzymes catalyzing the last step in the biosynthesis of two phytohormones, auxin and abscisic acid [88,89], both of which playing crucial roles in seed development and germination [8,90,91,92]. In conclusion, the potential longevity of seeds appears to be the most affected seed trait via Met oxidation in our study. As seed longevity is a very important aspect in seed science, the results presently described may contribute to further research in this direction. 

While a number of studies have demonstrated the role of redox regulation in seeds [42,59,93,94], the possible implication of reversible Met oxidation of specific proteins in modulating seed traits has only been partially recognized [30,42]. The biological functions of the presently characterized proteins unravel a major role of this post-translational modification in seed biology, particularly in relation to seed viability. Future research will concentrate on the large-scale characterization of the specific oxidized protein targets toward a better understanding of the impact of this protein modification on cellular functions and its involvement in ROS signaling in the seed aging process. We expect that the 2Dd-CNBr method will contribute to the discovery of novel protein elements of ROS-induced signal transduction pathways, notably concerning those proteins containing exposed Met residues that can be oxidized and reversibly reduced by the MSR system, and that appear to be potential candidates supporting such regulatory functions. Met oxidation can have several effects on protein structure through synergistic/antagonist interactions between different post-translational modifications. In this context, it is worth noting that studies in human cell lines revealed that the majority of proteins sulfoxidized after exposure to H_2_O_2_ were phosphoproteins [39]. Despite the progress in deciphering possible interactions between Met sulfoxidation and protein carbonylation or protein phosphorylation [27,95,96,97], the molecular mechanisms coupling oxidative signals to changes in phosphorylation in seeds still remain poorly understood. Interestingly, two of identified proteins containing oxidized Met and a phosphorylation site corresponded to an elongation factor and an inhibitor of serine proteases (Table 2), and were previously demonstrated to be substrates for MSRB1 [23] and periplasmic MSR [98], respectively. Hence, 2Dd-CNBr-based results might provide initial information toward a better understanding of the interplay between different types of post-translational protein modifications in targeted proteins. As the 2Dd-CNBr method allows for the characterization of Met oxidation at the proteome level, it is anticipated that its future application will not be limited only to individual seed proteins, but might be useful for a wide range of living organisms. Additionally, in combination with high-throughput gel-free proteomic approaches, this method may allow for further identifying the structural features underpinning this post-translational modification of proteins. Finally, the method presently described also has the potential for wider applications.

## 4. Materials and Methods

### 4.1. Plant Material

*Arabidopsis thaliana* seeds, accession Columbia, were used in all of the experiments. Seeds were first sown in a mixture of soil and vermiculite (9:1), and then placed in a growth chamber at 21 (±1) °C under a photoperiod of 16 h light/8 h dark (Fluorescent Tube compact Sylvania LYNX-LE 55W 860 6000 K). The light intensity was between 150 and 200 µmolm^−2^ s^−1^. After 12–15 d, the germinated seeds were transferred on soil/perlite/vermiculite (2:1:1) and the plants were grown in the same growth chamber as for germination. Siliques were harvested at maturity, dried at room temperature (approximately 22 °C) for 10 d, and the seeds were collected. To release dormancy, freshly harvested seeds were after-ripened at 20 °C (approximately 30% relative humidity) in the dark [99] and then stored at −30 °C.

### 4.2. Preparation of Soluble Proteins Extracts

Mature dry Arabidopsis seeds (200 mg) were ground in liquid nitrogen using a mortar and pestle. Soluble proteins were extracted from the resulting powder at 4 °C in 4 mL of an extraction buffer containing 250 mM Tris pH 7.5, 0.1% (*v*/*v*) antiprotease cocktail (Sigma-Aldrich, St. Louis, MO, USA), and 20 mM dithiothreitol (DTT) (Sigma-Aldrich). After 2 min at 4 °C, the homogenate was briefly centrifuged (10,000× *g*, 30 s, 4 °C), and the resulting supernatant was filtered through a syringe equipped with a polytetrafluoroethylene (PTFE) membrane filter unit (Minisart^®^ Sartorius, Göttingen, Germany; 25 mm, 0.2 µm pore size) to obtain the soluble protein extract. Proteins were precipitated in 10% (w/v) trichloroacetic acid (TCA) for 1 h at 4 °C. After centrifugation (20,000× *g*, 10 min, 4 °C), the precipitate was washed two times with 2 mL of 80% (*v*/*v*) acetone, 0.07% (*v*/*v*) 2-mercaptoethanol for 1 h at −20 °C, and then dried at room temperature for 5 min. The resulting pellet was solubilized in a twice concentrated Laemmli buffer [53]. The protein concentrations were measured using a 2-D Quant Kit (GE Healthcare). Bovine serum albumin was used as a standard.

### 4.3. In Gel Oxidation of Soluble Proteins and BSA with Hypochlorous Acid

Solutions of HOCl were freshly prepared in 0.1% (*w*/*v*) NaOH and standardized by absorbance measurements using ɛ_292_ (OCl^−^) = 350 M^−1^.cm^−1^ and ɛ_235_ (HOCl) = 100 M^−1^.cm^−1^. The hypochlorous acid solution was diluted into 250 mM Tris pH 7.5 to obtain a final HOCl concentration of 0.5 mM.

### 4.4. D-Diagonal Electrophoresis, Gel Staining, and Image Analysis

One-dimensional SDS-PAGE of 40-µg soluble Arabidopsis protein extracts or 100 ng BSA (Sigma-Aldrich) was performed using 10% (*w*/*v*) polyacrylamide resolving gels [53]. Electrophoresis was performed in a Mini Protean (Bio-Rad, Hercules, CA, USA) using a Tris-Glycine-SDS running buffer (Bio-Rad), at 200 V and 40 mA. After completion of the first dimension, the gel lanes (referred later to as gel strips) were cut out, washed 1 min in deionized sterile water, and briefly soaked in 250 mM Tris pH 7.5 or in 0.5 mM HOCl solution for the control and the oxidized conditions, respectively. The gel strips were washed 1 min in water and then incubated with agitation for 30 min in a cleavage buffer (500 mM CNBr (Sigma-Aldrich) diluted in 70% (*v*/*v*) formic acid) under a fume hood at 22 ± 2 °C. The gel strips were briefly rinsed and washed twice for 5 min in deionized sterile water. Then, the gel strips were equilibrated twice for 10 min in an equilibration solution containing 6 M urea, 20% (*v*/*v*) glycerol, 2% (*w*/*v*) SDS, and 0.375 M Tris pH 8.8. DTT (100 mM) was added to the first equilibration solution, and iodoacetamide (125 mM) was added to the second one. Then, gel strips were placed on top of 4% stacking gels onto 15% resolving gels. A small amount of 1% agar solution was used to fuse the gel strip to the 2D gel. The second-dimension separation was performed under the same conditions as the first-dimension run. A silver staining method compatible with the analysis by mass spectrometry [100] was used to stain the gels. For each treatment analyzed, 2Dd gel experiments were done three times, corresponding to biological and technical repeats. Stained gels (second dimension) were imaged using a ViewPix 900 Imaging System (Biostep^®^ GMBH, Burkhardtsdorf Germany) and analyzed using Image Master Platinum software (GE Healthcare).

### 4.5. Protein Identification by Mass Spectrometry

In-gel digestion was performed with the Progest system (Genomic Solution, Bengaluru, India) according to a standard trypsin protocol. Gel pieces were washed twice by successive separate baths of 10% (*v*/*v*) acetic acid, 40% (*v*/*v*) ethanol, and acetonitrile (ACN). They were then washed twice with successive baths of 25 mM NH_4_CO_3_ and ACN. Digestions were subsequently performed for 6 h at 37 °C with 125 ng of modified trypsin (Promega) dissolved in 20% (*v*/*v*) methanol and 20 mM NH_4_CO_3_. The peptides were extracted successively with 2% (*w*/*v*) trifluoroacetic acid (TFA) and 50% (*v*/*v*) can and then with ACN. Peptide extracts were dried in a vacuum centrifuge and suspended in 20-µL of 0.05% (*v*/*v*) TFA, 0.05% (*v*/*v*) HCOOH, and 2% (*v*/*v*) ACN. HPLC was performed using a NanoLC-Ultra system (Eksigent, Dublin, OH, USA). A 4-µL sample was loaded at 7.5 µL/min^−1^ on a precolumn cartridge (stationary phase: BIOSPHERE C18, 5 µm; column: 100 µm i.d. 2 cm; NanoSeparations) and desalted with 0.1% (*v*/*v*) HCOOH. After 3 min, the precolumn cartridge was connected to a separating PepMap C18 column (stationary phase: BIOSPHERE C18, 3 µm; column: 75 µm i.d. 150 mm; NanoSeparations). Buffers were 0.1% (*v*/*v*) HCOOH in water (A) and 0.1% HCOOH (*v*/*v*) in ACN (B). Peptide separation was achieved with a linear gradient from 5 to 30% B for 11 min at 300 nl/min^−1^. Including the regeneration step at 95% B and the equilibration step at 95% A, each run took 25 min. The eluted peptides were analyzed on-line with a Q-Exactive mass spectrometer (Thermo Electron, Whaltam, MA, USA) using a nano-electrospray interface (non-coated capillary probe, 10 µ i.d.; New Objective). The Xcalibur 2.1 interface was used to monitor data-dependent acquisition of peptide ions. This included full MS scan covering a 300-to-1400 range of mass-to-charge ratio (m/z) with a resolution of 70,000 and a MS/MS step (normalized collision energy: 30%; resolution: 17,500). The MS/MS step was reiterated for the five major ions detected during full MS scan. Dynamic exclusion was set to 45 s. A database search was performed with XTandem (version 2012.10.01.1) (http://www.thegpm.org/TANDEM/, accessed on 20 November 2012). Enzymatic cleavage was declared as a trypsin and CNBr digestion with two possible miscleavages. Cys carboxyamidomethylation was set to static modifications. The Met were searched for oxidation, homoserine, and homoserine lactone as possible modifications. Precursor mass and fragment mass tolerance were 10 ppm and 0.02 Th, respectively. A refinement search was added with similar parameters, except that semi-trypsic peptide and possible N-ter proteins acetylation were searched. The Arabidopsis Information Resource (http://www.arabidopsis.org/, accessed on 20 November 2012) database (32825 entries, version 8) and a contaminant database (trypsin, keratins) were used. Only peptides with an E value smaller than 0.1 were reported. The identified proteins were filtered and grouped using XTandem Pipeline (http://pappso.inra.fr/bioinfo/xtandempipeline/, accessed on 20 November 2012) according to: (i) A minimum of two different peptides was required with an E value smaller than 0.05, and (ii) a protein E value (calculated as the product of unique peptide E values) smaller than 10^−4^. In the case of identification with only two or three MS/MS spectra, similarity between the experimental and the theoretical MS/MS spectra was visually checked. To take redundancy into account, proteins with at least one peptide in common were grouped. This allowed for grouping proteins of a similar function. Within each group, proteins with at least one specific peptide relative to the other members of the group were reported as sub-groups. 

### 4.6. Bioinformatic Tools

WebLogo 3.3 (http://weblogo.berkeley.edu/logo.cgi, accessed on 1 September 2015) was used to generate a sequence logo [101]. PhosPhAt 4.0 (http://phosphat.mpimp-golm.mpg.de/phosphat.html, accessed on 4 May 2020), a database of phosphorylation sites in Arabidopsis proteins, was used to predict the phosphorylation sites in the identified peptides [102]. The search for the presence of amino acids known to be potentially modified, such as Pro, Arg, and Glu, or Ser, Thr, and Tyr, in the case of carbonylation was performed according to Rao and Moller [93]. Jmol: an open-source Java viewer for chemical structures in 3D (http://www.jmol.org/, accessed on 1 September 2015) was used as a viewer of the molecular structures. RaptorX (http://raptorx.uchicago.edu/StructurePrediction, accessed on 1 September 2015) was used for the automated protein secondary structure prediction and template-based tertiary structure modeling [103].

## Figures and Tables

**Table 1:** Proteins from mature Arabidopsis seeds containing oxidized Met with a specified position of Met (M). The Arabidopsis Information Resource (TAIR) accession (acc.) number, protein name, identified peptide sequences, and Met position in protein sequences are indicated. For further details on these protein, see Table S4.

Table	Protein Name	Peptide Sequence	Met Position
AT2G42560.1	Late embryogenesis abundant domain	GSNMPVSDEGEGETK DQEMHQGGEEEKQPGFVSGAR THEHGTTDPDYMR	M65 M487 M628
AT1G07920.1	Elongation factor 1-alpha 3	VETGMIKPGMVVTFAPTGLTTEVK PGMVVTFAPTGLTTEVK MTPTKPMVVETFSEYPPLGR	M259, 264 M264 M398
AT4G24620.1	Glucose-6-phosphate isomerase 1, chloroplastic	VGFTDEFVAEMEPR YLQQLVMESLGK	M107 M391
AT2G36640.1	Late embryogenesis abundant protein	LTMPSDIVEETR	M382
AT1G21750.1	Protein disulfide isomerase-like 1–1	LSGSEFDSFMAIAEK FPKLSGSEFDSFMAIAEK	M180 M180
AT3G17240.1	Dihydrolipoyl dehydrogenase 2, mitochondrial	VSSVEVDLPAMLAQK	M124
AT3G22500.1	Late embryogenesis abundant protein in group 5	GGPAAVMQSAATTNIR	M58
AT3G48990.1	4-Coumarate-CoA ligase-like 10	SSNPLPEEGPHKPGSVGKP VGQEMAILNEK	M345
AT3G53040.1	Late embryogenesis abundant protein-like	TTTTEPERPGLIGSVMK	M59
AT3G54400.1	Aspartyl protease family protein	ASGTSLPAQGLMGLGR	M209
AT4G12290.1	Amine oxidase	VGLSGILMVK	M491
AT5G20960.1	Indole-3-acetaldehyde oxidase	VPAVYAVNMR	M1046
AT5G44120.3	12S seed storage protein CRA1	DMHQKVEHIR	M138
AT5G52300.1	Low-temperature-induced 65 kDa protein	MESQLTRPYGHEQAEEPIR	M1
AT2G31670.1	Uncharacterized protein	DLSEMEAVDAQK	M223
AT1G54100.1	Aldehyde dehydrogenase family 7 member B4	VGSMVQQTVNAR	M251
AT1G77510.1	Protein disulfide isomerase-like 1–2	LSGDEFDSFMALAEK	M179
AT2G36530.1	Bifunctional enolase 2/transcriptional activator	VVIGMDVAASEFYSEDK	M249
AT1G47710.1	Cysteine protease inhibitor/ serine-type endopeptidase inhibitor	ESISLQNQVSMNLAK	M15

**Table 2:** Identified proteins containing oxidized Met and a phosphorylation site. Amino acids (Ser, Thr, and Tyr) surrounding MetO of proteins listed in this table have been located in the corresponding 3D structures using Jmol software. Phosphorylation sites were predicted using PhosPhAt 4.0 tool. For AT5G44120.3 (12S seed storage protein CRA1) the phosphorylation site was confirmed experimentally. The Arabidopsis Information Resource (TAIR) accession (acc.) number; protein name; and Met, Ser, Thr, and Tyr position in the protein sequences are indicated.

TAIR acc.	Protein Name	Met Position	Ser, Thr, and Tyr Position *
AT1G07920.1	Elongation factor 1-alpha 3	M264	S315
AT4G12290.1	Amine oxidase	M491	Y243
AT5G20960.1	Indole-3-acetaldehyde oxidase	M1046	Y1179
AT5G44120.3	12S seed storage protein CRA1	M138	T115 **
AT1G54100.1	Aldehyde dehydrogenase family 7 member B4	M251	S250
AT1G47710.1	Cysteine protease inhibitor/serine-type endopeptidase inhibitor	M15	Y239

* In the 3D structure as revealed by using Jmol software (http://www.jmol.org accessed on 1 September 2015). ** Phosphorylation site predicted by PhosPhat 4.0 was experimentally confirmed in Wan et al. [59].

## Data Availability

The data presented in this study are available upon request from the corresponding author.

## Supplementary Materials

Supplementary Materials can be found at https://www.mdpi.com/article/10.3390/plants11040569/s1. Table S1: List of 986 identified peptides (control 2Dd-CNBr). Table S2. List of identified proteins considered as abundant. Table S3. List of peptides containing oxidized or reduced Met (control 2Dd-CNBr). Table S4. List of identified proteins containing oxidized Met. Table S5. List of 378 identified peptides (oxidized 2Dd-CNBr). Table S6. List of the 5968 identified peptides from two independent SDS-PAGE separations. Table S7. Data used for the search of 3D models. Figure S1. WebLogo graphs. Figures S2–S14. 3D models of identified proteins Figure S2: AT3G48990; Figure S3: AT5G20960; Figure S4: AT1G54100; Figure S5: AT3G54400; Figure S6: AT1G21750; Figure S7: AT1G77510; Figure S8: AT4G12290; Figure S9: AT1G07920; Figure S10: AT2G36530; Figure S11: AT3G17240; Figure S12: AT4G24620; Figure S13: AT2G31670; Figure S14: AT1G47710. Figure S15. 3D environment (3–7 Å) of oxidized Met. Figure S16. Disorder prediction for AT5G44120.3.
