# Supplementary material for: Are Methionine Sulfoxide-Containing Proteins Related to Seed Longevity? A Case Study of Arabidopsis thaliana Dry Mature Seeds Using Cyanogen Bromide Attack and Two-Dimensional-Diagonal Electrophoresis
Source: Plants (Basel). 2022 Feb 21;11(4):569. doi: 10.3390/plants11040569 (PMC8875303; doi:10.3390/plants11040569)

Figure S1. WebLogo 3.3 (Crooks et al. 2004) was used to create logo graphs colored by (A) hydrophobicity, (B) charge, (C) chemistry using color code given below. Analyses were made using 15 amino acids localized linear before and after oxidized methionine (position "O" in graphs) of all proteins identified by mass spectrometry and listed in Table 1.

◎ Hydrophobicity (AA default):

Hydrophilic	RKDENQ	blue
Neutral	SGHTAP	green
Hydrophobic	YVMCLFIW	black

◎ Charge (AA):

Positive	KRH	blue
Negative	DE	red

◎ Chemistry (AA):

Polar	G,S,T,Y,C	green
Neutral	Q,N	purple
Basic	K,R,H	blue
Acidic	D,E	red
Hydrophobic	A,V,L,I,P,W,F,M	black

A)

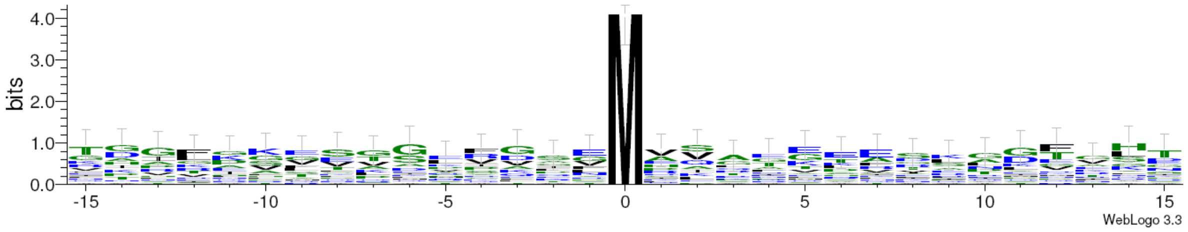

B)

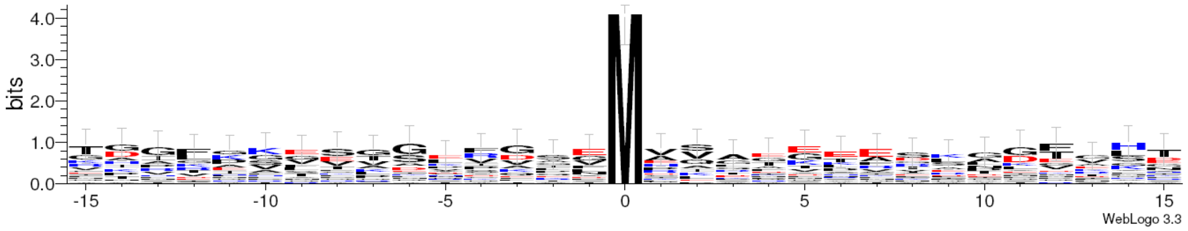

C)

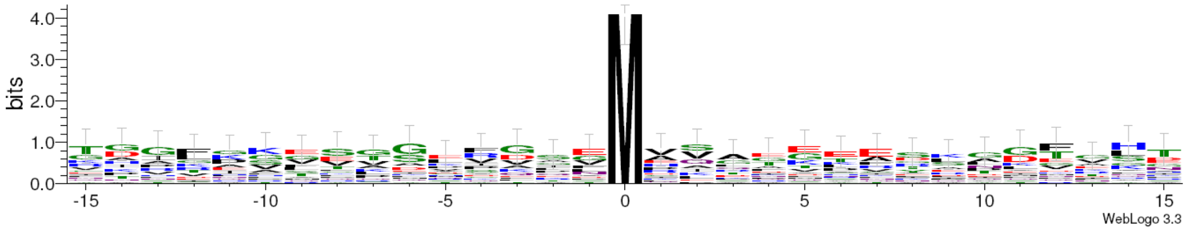

Supplement: Supplementary file 1 [file plants-11-00569-s001.zip › plants-1599886(1)/Figure S1.pdf]
