# Supplementary material for: Are Methionine Sulfoxide-Containing Proteins Related to Seed Longevity? A Case Study of Arabidopsis thaliana Dry Mature Seeds Using Cyanogen Bromide Attack and Two-Dimensional-Diagonal Electrophoresis
Source: Plants (Basel). 2022 Feb 21;11(4):569. doi: 10.3390/plants11040569 (PMC8875303; doi:10.3390/plants11040569)

Figure S15. Three dimensional environment of identified oxidized Met within a distance of 3-7 Å. Vertical bars represent the proportion of very hydrophobic (Phe, Ile, Trp, Leu, Val, Met), hydrophobic (Tyr, Cys, Ala), neutral (Thr, His, Gly, Ser, Gln) and hydrophilic (Arg, Lys, Asn, Glu, Pro, Asp) amino acids at a distance of 3, 4, 5, 6 and 7 Å of oxidized Met.

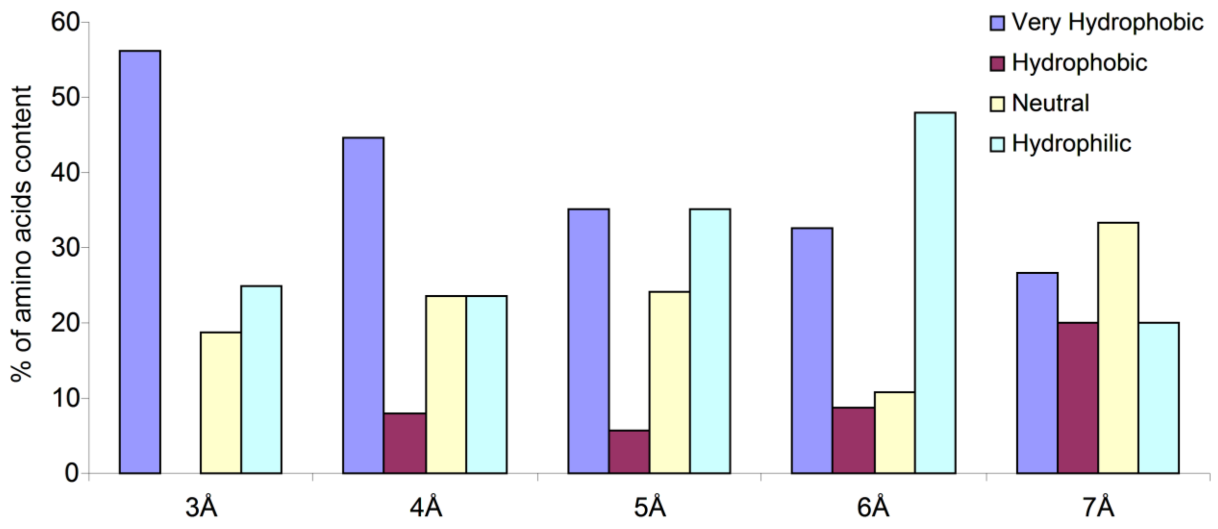

Supplement: Supplementary file 1 [file plants-11-00569-s001.zip › plants-1599886(1)/Figure S15.pdf]
