# Supplementary material for: Are Methionine Sulfoxide-Containing Proteins Related to Seed Longevity? A Case Study of Arabidopsis thaliana Dry Mature Seeds Using Cyanogen Bromide Attack and Two-Dimensional-Diagonal Electrophoresis
Source: Plants (Basel). 2022 Feb 21;11(4):569. doi: 10.3390/plants11040569 (PMC8875303; doi:10.3390/plants11040569)

Figure S16. A disorder prediction for the entire target sequence AT5G44120.3 was done by using Raptorx server (http://raptorx.uchicago.edu/StructurePrediction). The color diagram shows the disorder prediction; residues in a disorder segment were marked in red or non-disorder segment were marked in blue, red arrow shows the localization of oxidized M138.

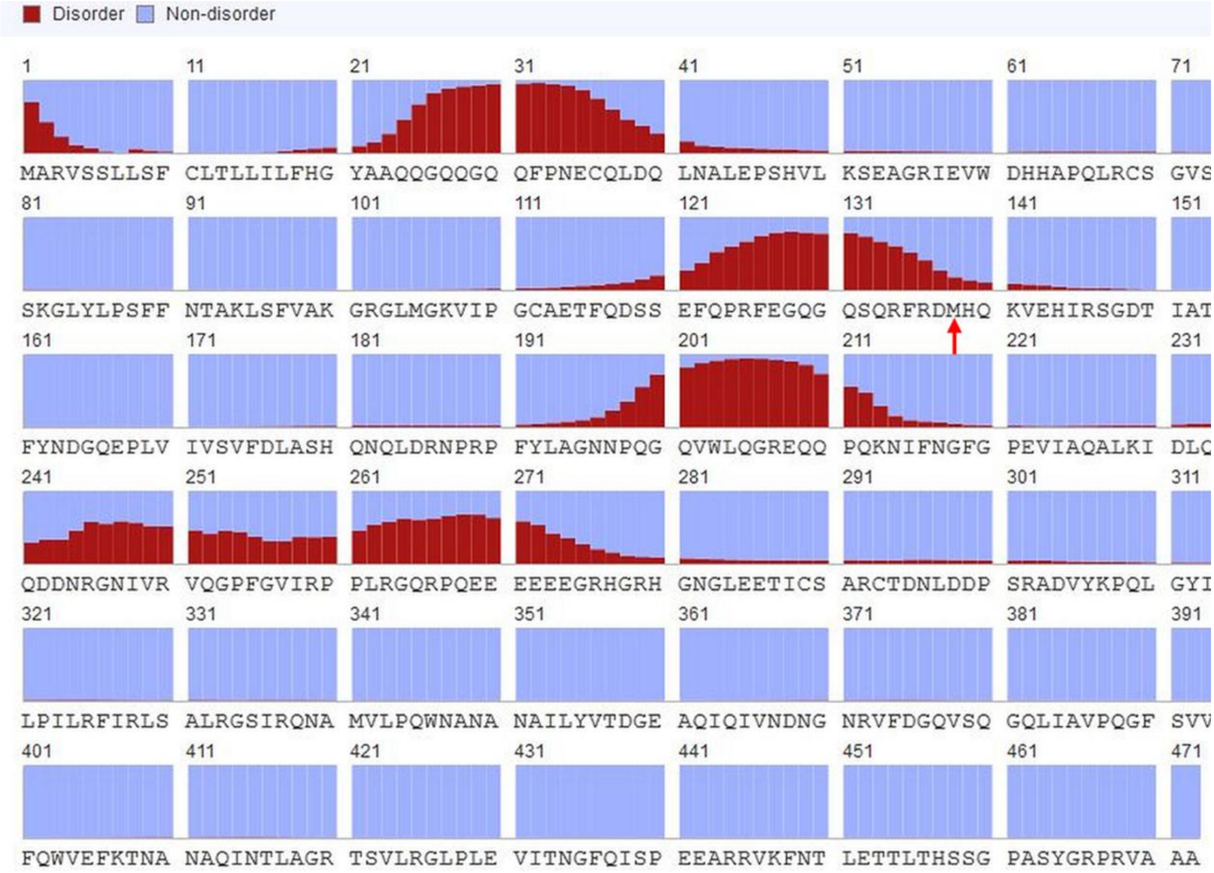

Supplement: Supplementary file 1 [file plants-11-00569-s001.zip › plants-1599886(1)/Figure S16.pdf]
