# Supplementary material for: Are Methionine Sulfoxide-Containing Proteins Related to Seed Longevity? A Case Study of Arabidopsis thaliana Dry Mature Seeds Using Cyanogen Bromide Attack and Two-Dimensional-Diagonal Electrophoresis
Source: Plants (Basel). 2022 Feb 21;11(4):569. doi: 10.3390/plants11040569 (PMC8875303; doi:10.3390/plants11040569)

3D models of identified proteins using Jmol software

Figure S2. 3D model of identified protein assigned as AT3G48990 using Jmol software. Green dotted circles show oxidized Met position, blue areas depict solvent-accessible surface.

AT3G48990

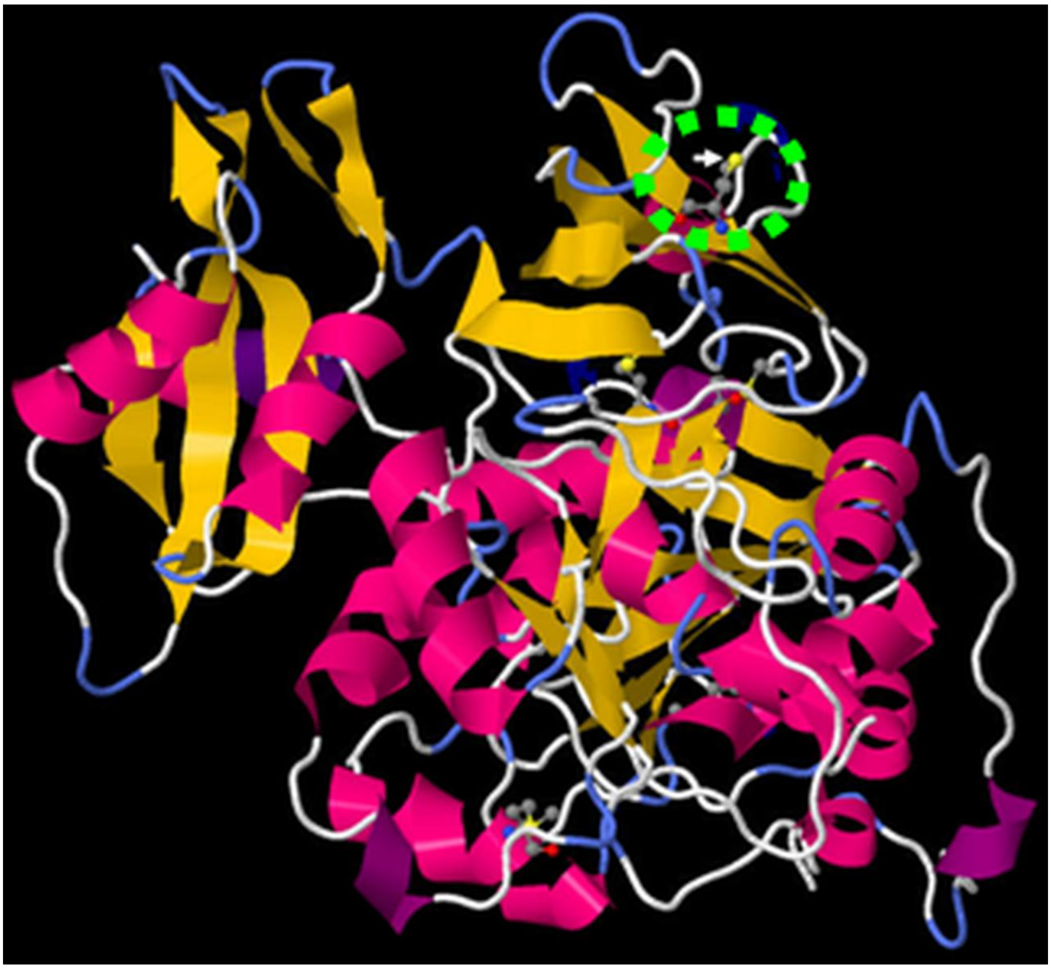

Figure S3. 3D model of identified protein assigned as AT5G20960 using Jmol software. Green dotted circles show oxidized Met position, blue areas depict solvent-accessible surface.

AT5G20960

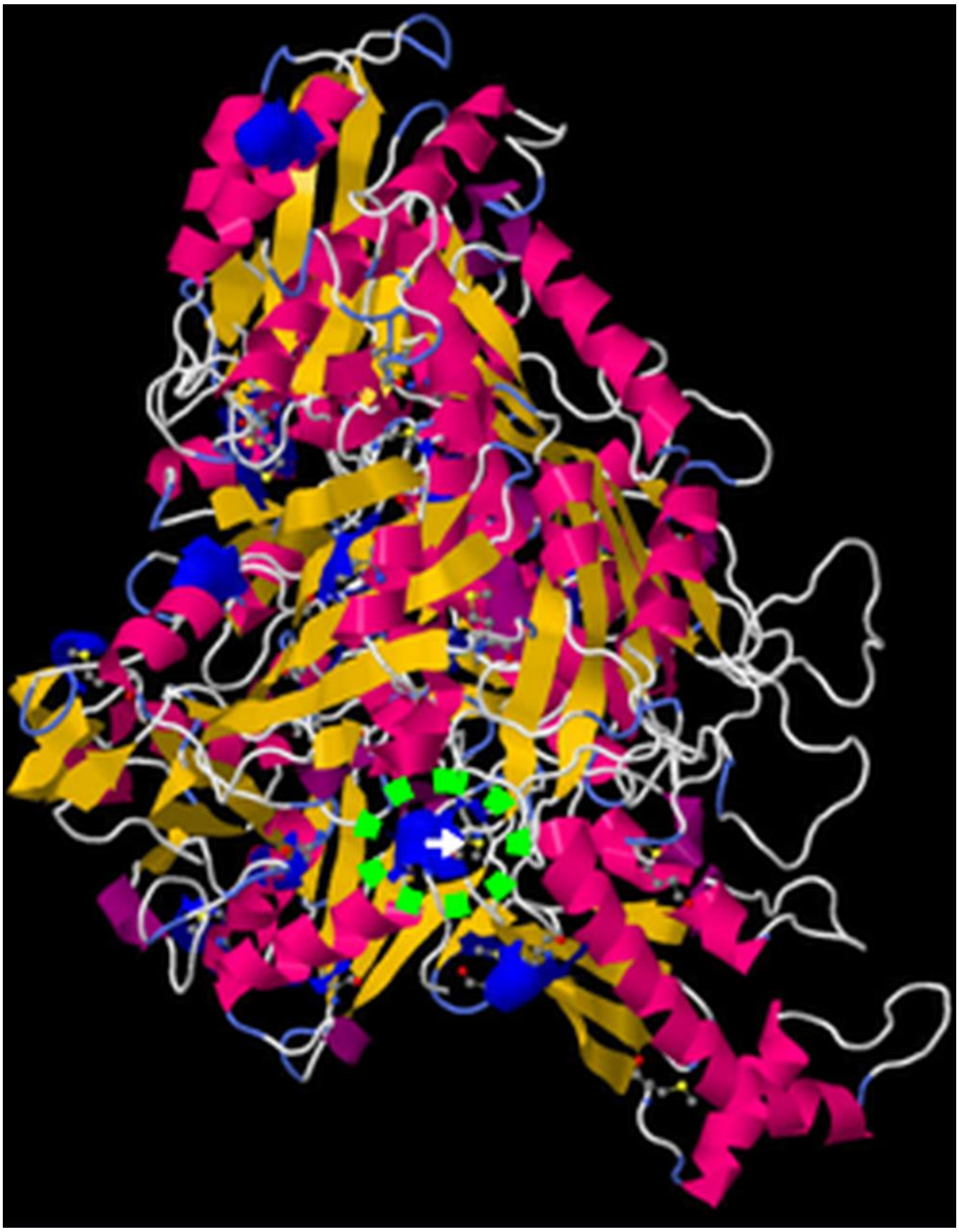

Figure S4. 3D model of identified protein assigned as AT1G54100 using Jmol software. Green dotted circles show oxidized Met position, blue areas depict solvent-accessible surface.

AT1G54100

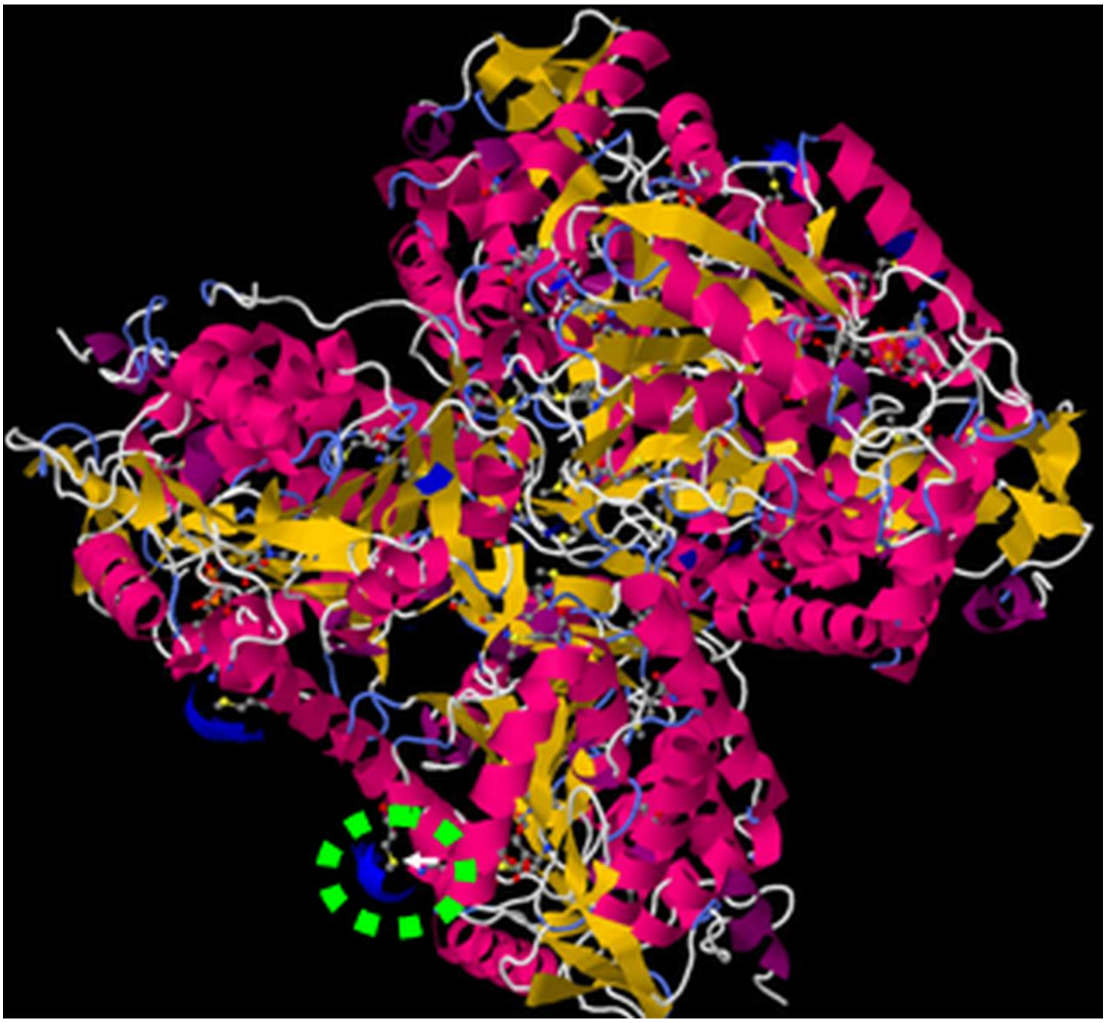

Figure S5. 3D model of identified protein assigned as AT3G54400 using Jmol software. Green dotted circles show oxidized Met position, blue areas depict solvent-accessible surface.

AT3G54400

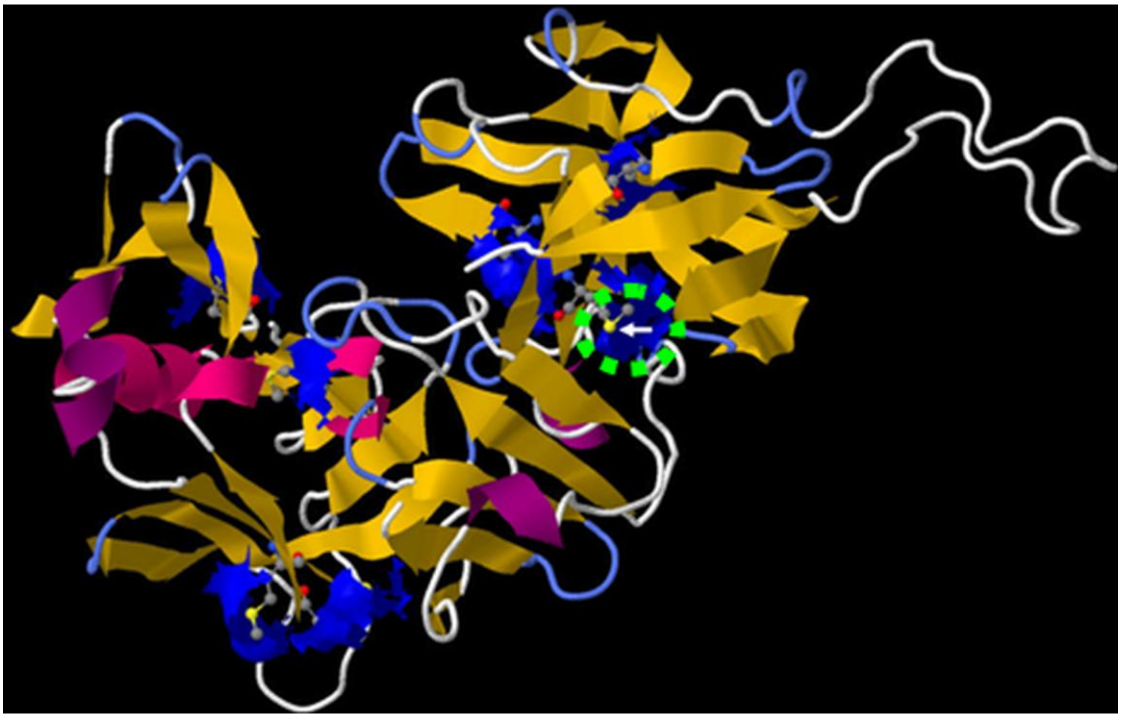

Figure S6. 3D model of identified protein assigned as AT1G21750 using Jmol software. Green dotted circles show oxidized Met position, blue areas depict solvent-accessible surface.

AT1G21750

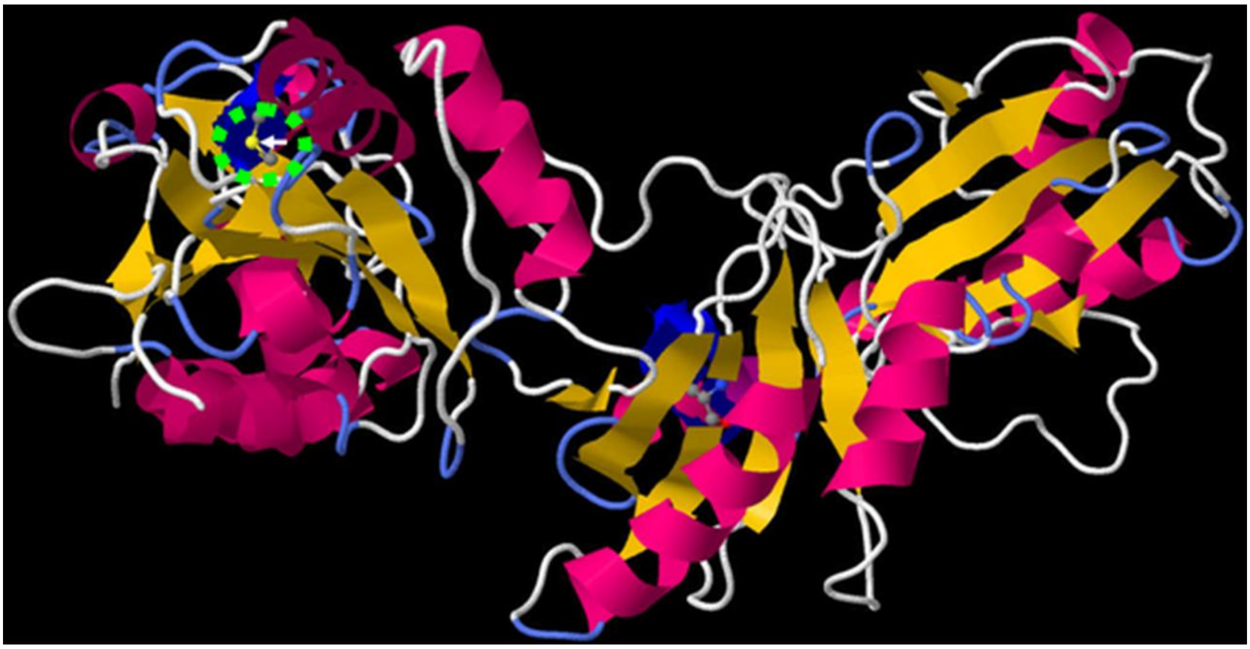

Figure S7. 3D model of identified protein assigned as AT1G77510 using Jmol software. Green dotted circles show oxidized Met position, blue areas depict solvent-accessible surface.

AT1G77510

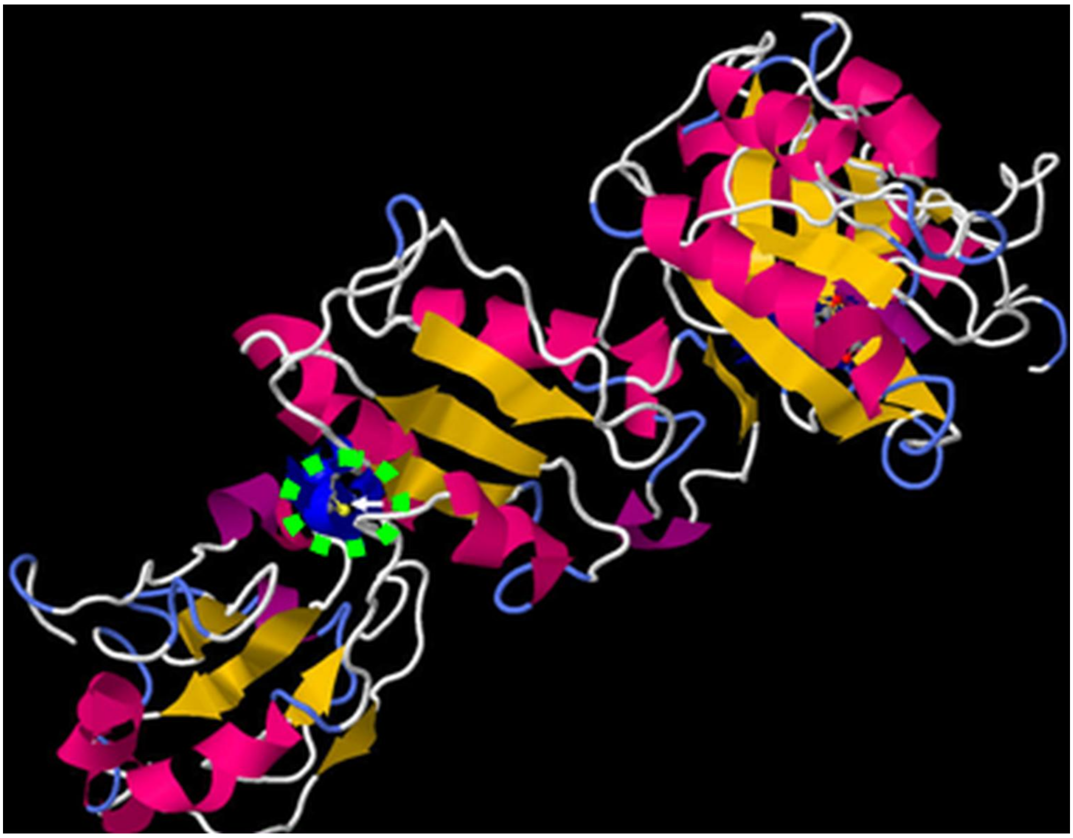

Figure S8. 3D model of identified protein assigned as AT4G12290 using Jmol software. Green dotted circles show oxidized Met position, blue areas depict solvent-accessible surface.

AT4G12290

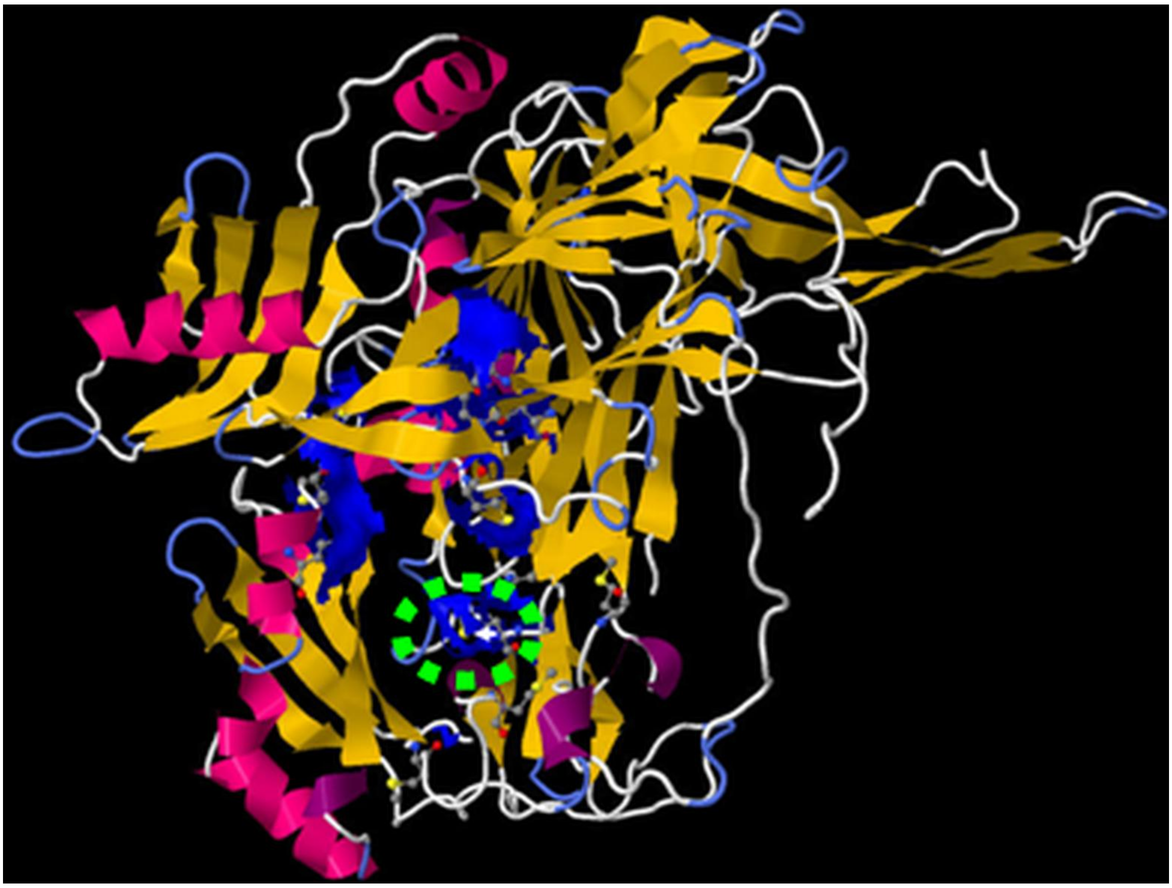

Figure S9. 3D model of identified protein assigned as AT1G07920 using Jmol software. Three methionine (M) residues are visualized at positions A: M259 and M264, B: M398. Green dotted circles show oxidized Met position, blue areas depict solvent-accessible surface.

AT1G07920

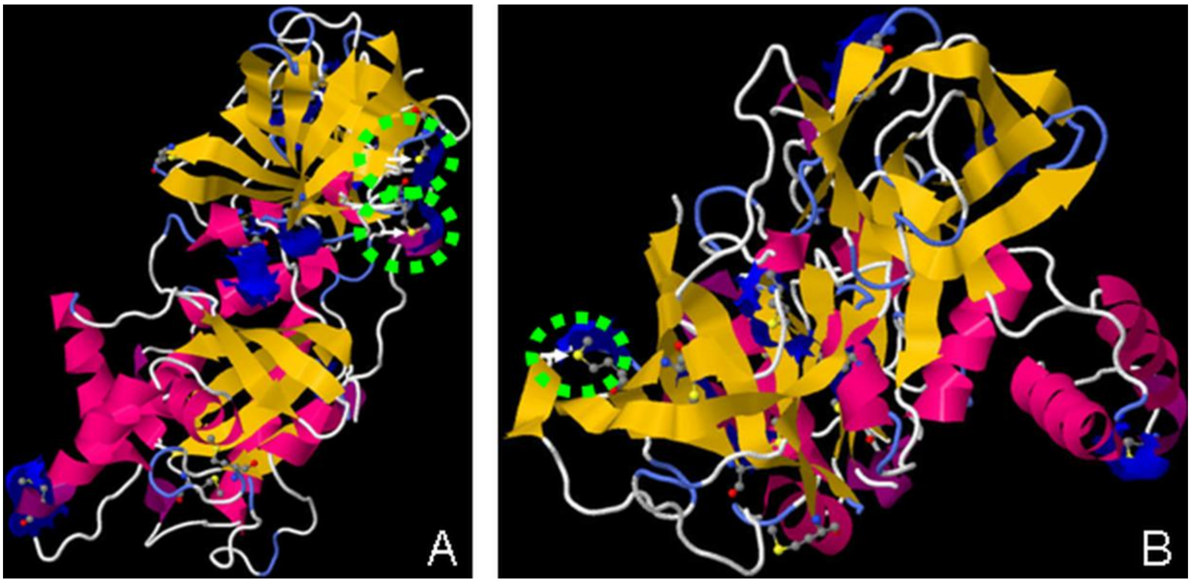

Figure S10. 3D model of identified protein assigned as AT2G36530 using Jmol software. Green dotted circles show oxidized Met position, blue areas depict solvent-accessible surface.

AT2G36530

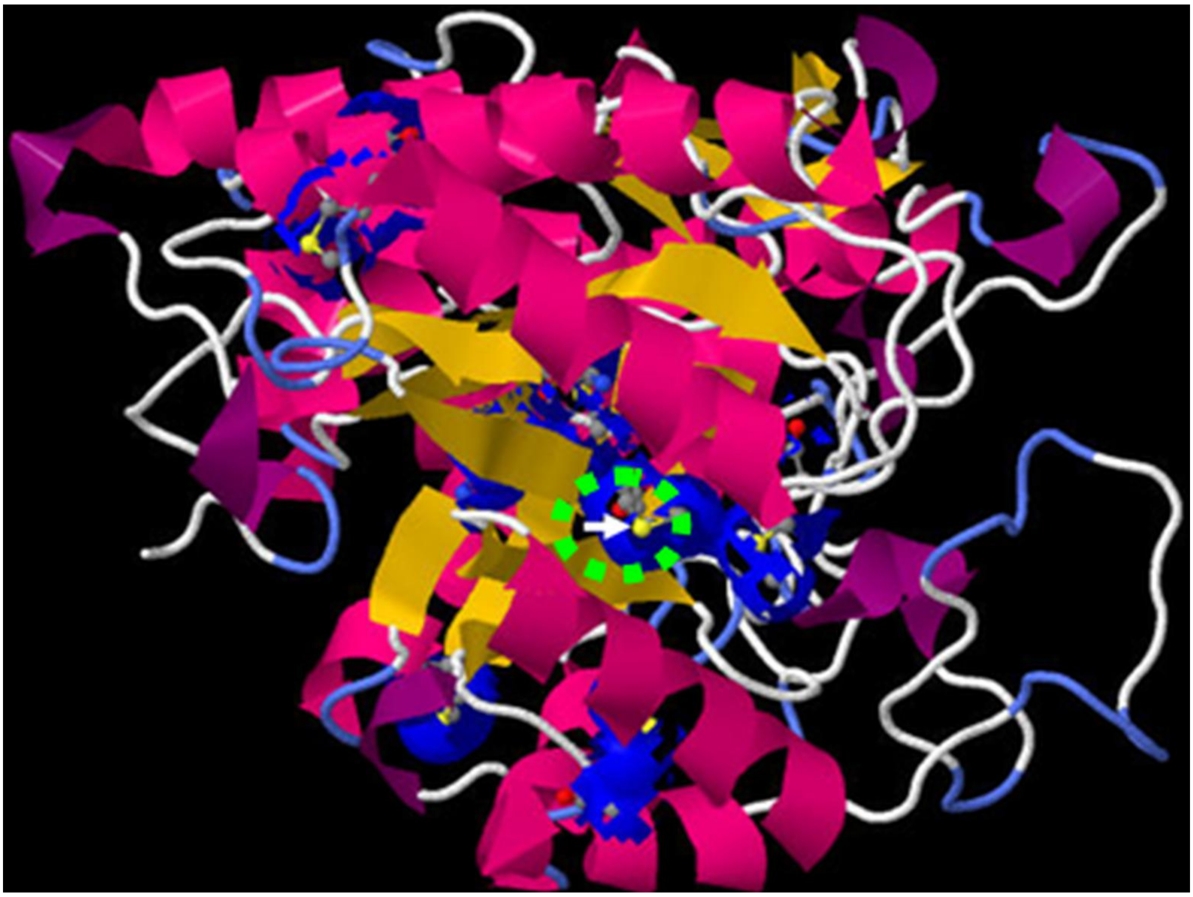

Figure S11. 3D model of identified protein assigned as AT3G17240 using Jmol software. Green dotted circles show oxidized Met position, blue areas depict solvent-accessible surface.

AT3G17240

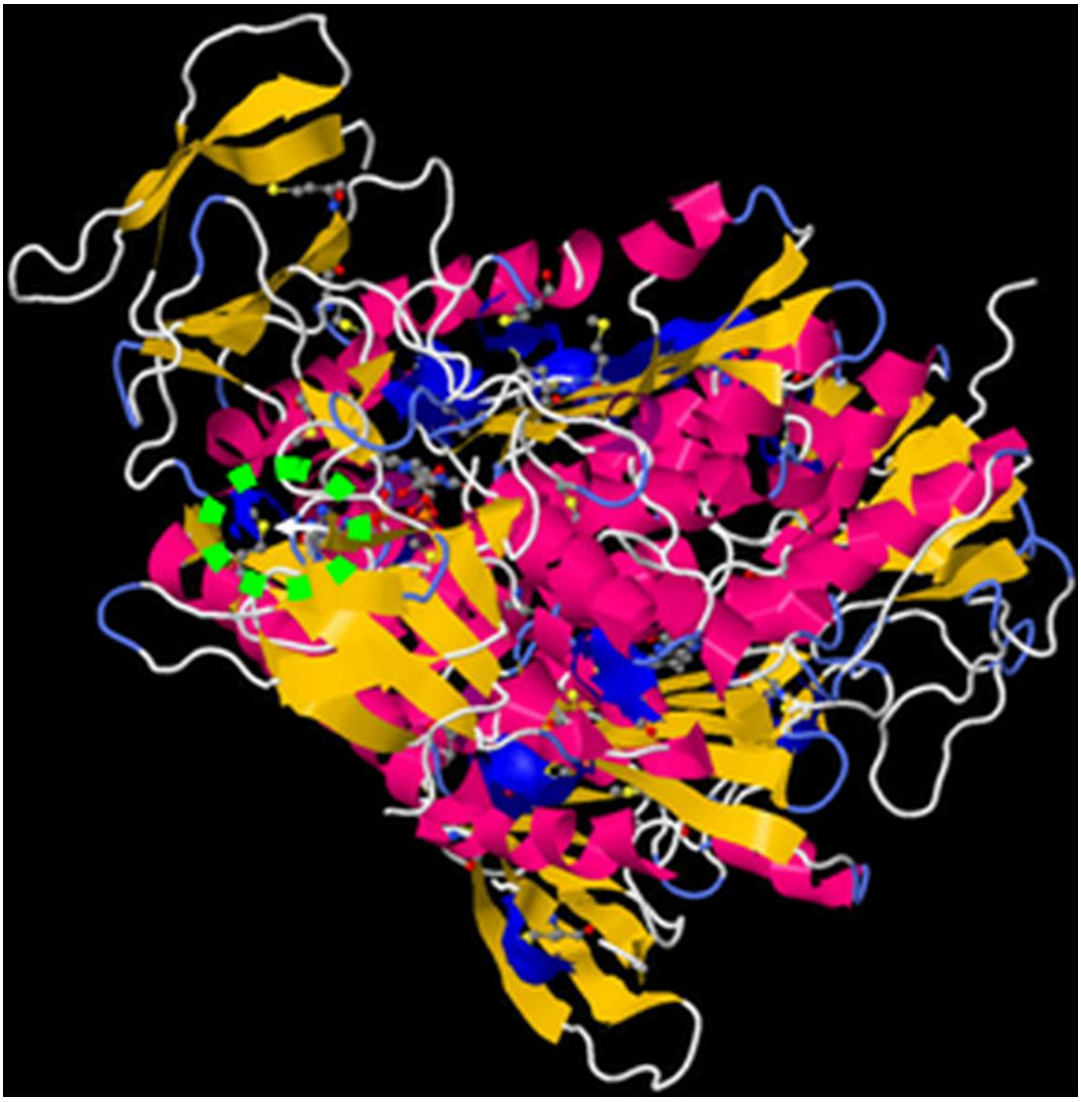

Figure S12. 3D model of identified protein assigned as AT4G24620 using Jmol software. Two methionine (M) residues are visualized at positions A: M107, B: M391. Green dotted circles show oxidized Met position, blue areas depict solvent-accessible surface.

AT4G24620

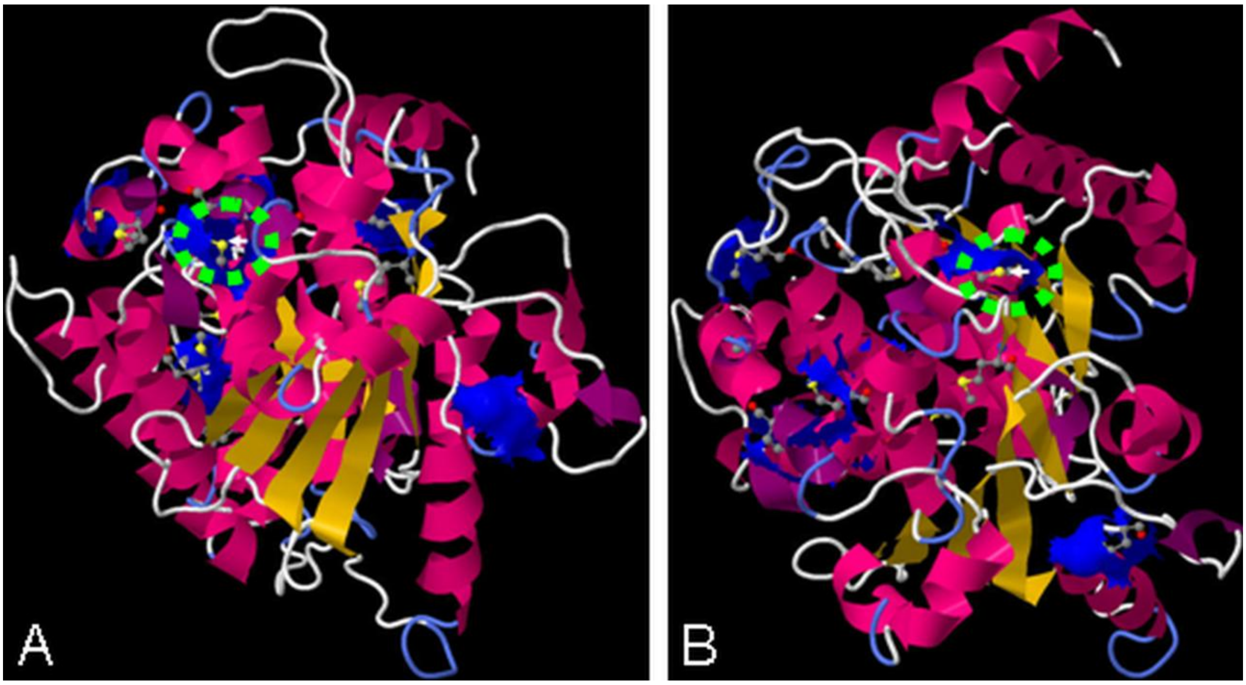

Figure S13. 3D model of identified protein assigned as AT2G31670 using Jmol software. Green dotted circles show oxidized Met position, blue areas depict solvent-accessible surface.

AT2G31670

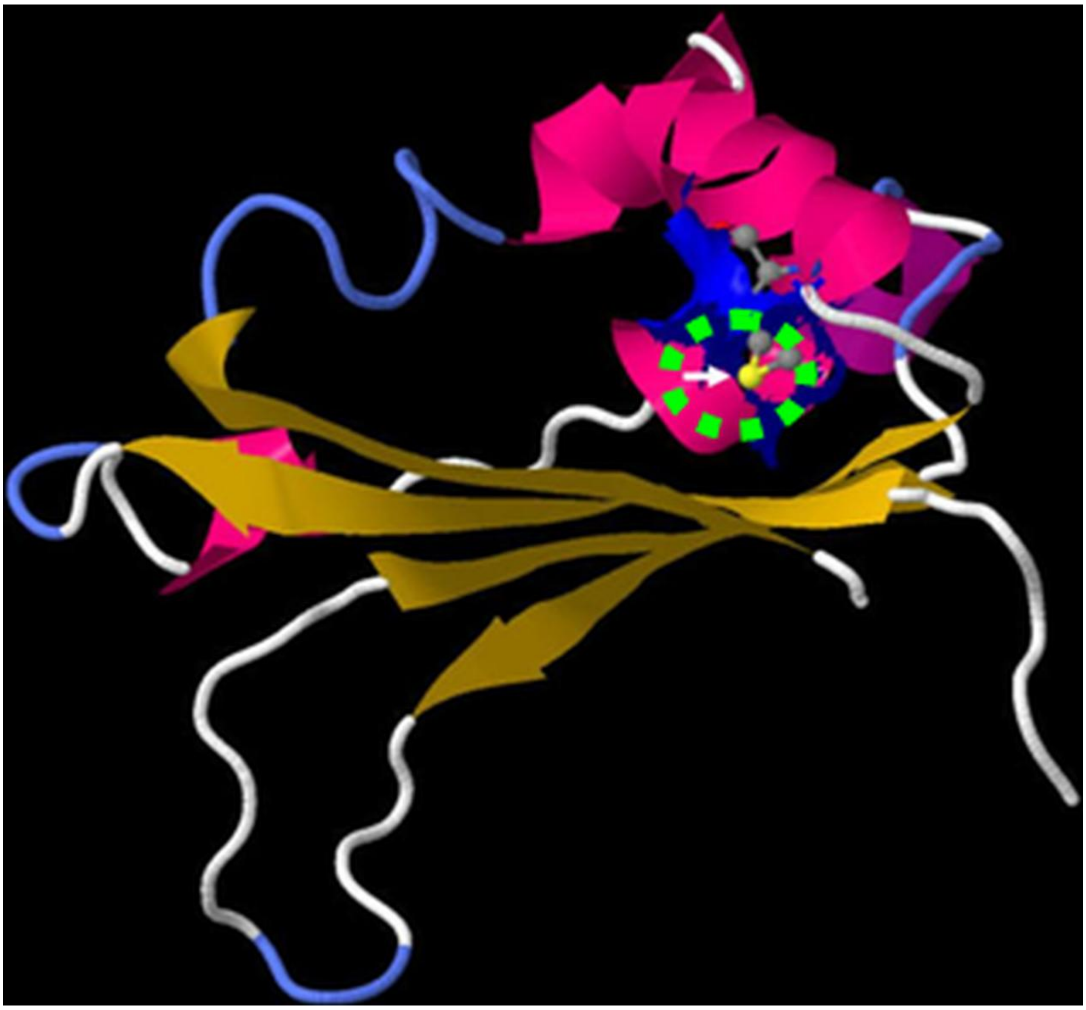

Figure S14. 3D model of identified protein assigned as AT1G47710 using Jmol software. Green dotted circles show oxidized Met position, blue areas depict solvent-accessible surface.

AT1G47710

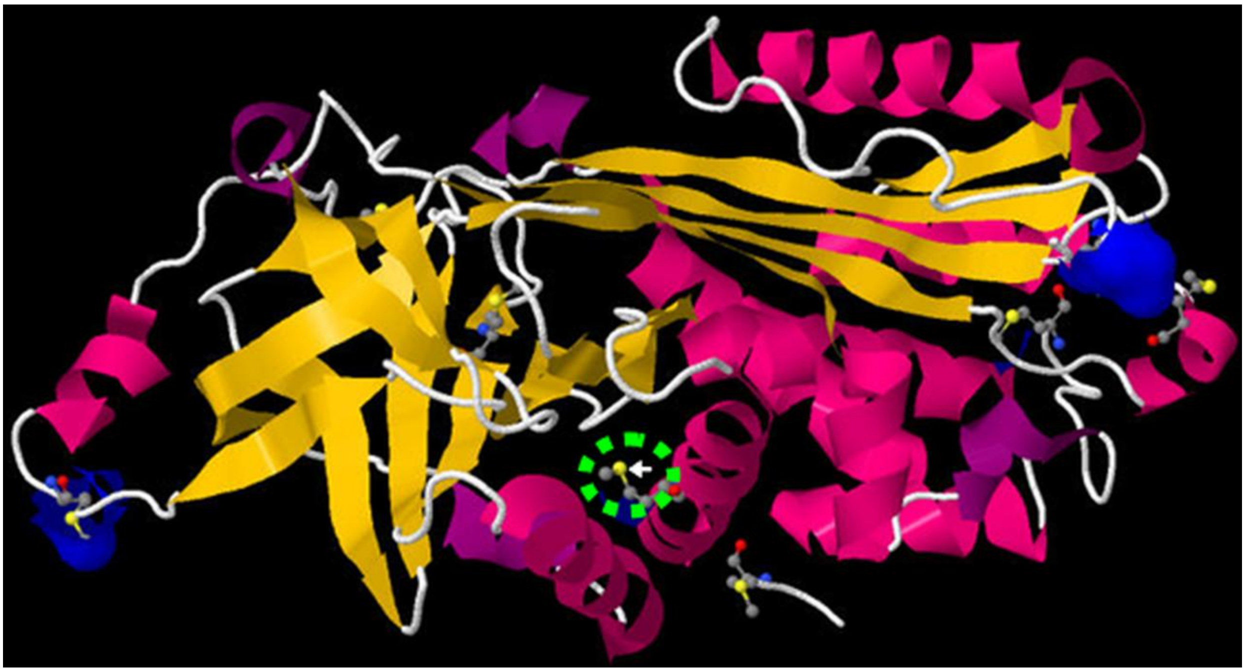

Supplement: Supplementary file 1 [file plants-11-00569-s001.zip › plants-1599886(1)/Figures S2-S14.pdf]
